# Deleted in Liver Cancer 1 (DLC1) Negatively Regulates Rho/ROCK/MLC Pathway in Hepatocellular Carcinoma

**DOI:** 10.1371/journal.pone.0002779

**Published:** 2008-07-23

**Authors:** Carmen Chak-Lui Wong, Chun-Ming Wong, Frankie Chi-Fat Ko, Lo-Kong Chan, Yick-Pang Ching, Judy Wai-Ping Yam, Irene Oi-lin Ng

**Affiliations:** Department of Pathology, SH Ho Foundation Research Laboratory and Jockey Club Clinical Research Centre, The University of Hong Kong, Hong Kong, China; University of Birmingham, United Kingdom

## Abstract

**Aims:**

Deleted in liver cancer 1 (DLC1), a member of RhoGTPase activating protein (GAP) family, is known to have suppressive activities in tumorigenicity and cancer metastasis. However, the underlying molecular mechanisms of how DLC1 suppresses cell motility have not been fully elucidated. Rho-kinase (ROCK) is an immediate down-stream effector of RhoA in mediating cellular cytoskeletal events and cell motility. In the present study, we aimed to investigate the effects of DLC1 on Rho/ROCK signaling pathway in hepatocellular carcinoma (HCC).

**Methodology/Principal Findings:**

We demonstrated that DLC1 negatively regulated ROCK-dependent actomyosin contractility. From immumofluorescence study, we found that ectopic expression of DLC1 abrogated Rho/ROCK-mediated cytoskeletal reorganization including formation of stress fibers and focal adhesions. It also downregulated cortical phosphorylation of myosin light chain 2 (MLC2). These inhibitory events by DLC1 were RhoGAP-dependent, as RhoGAP-deficient mutant of DLC1 (DLC1 K714E) abolished these inhibitory events. In addition, from western study, DLC1 inhibited ROCK-related myosin light chain phosphatase targeting unit 1 (MYPT1) phosphorylation at Threonine 853. By examining cell morphology under microscope, we found that ectopic expression of dominant-active ROCK released cells from DLC1-induced cytoskeletal collapse and cell shrinkage.

**Conclusion:**

Our data suggest that DLC1 negatively regulates Rho/ROCK/MLC2. This implicates a ROCK-mediated pathway of DLC1 in suppressing metastasis of HCC cells and enriches our understanding in the molecular mechanisms involved in the progression of hepatocellular carcinoma.

## Introduction

Cell migration involves cycles of steps which begin from the formation of cell protrusion at the leading edge. At the sites of protrusion, focal adhesions are formed to attach the cytoskeleton, mainly actin and myosin, to the extracellular matrix. The cytoskeleton generates tension which results in actomyosin contractility to translocate the cell body. Finally, the tension releases the adhesions from the cell's trailing edge [1]. Deregulation in any of the steps involved in cell migration can result in aberrant cell movement and, in cancer cells, metastasis [2]. Hepatocellular carcinoma (HCC) is one of the most prevalent cancers worldwide. Intrahepatic metastasis is the leading cause of mortality in patients with this cancer. Therefore, we believe a better understanding of the molecular mechanisms regulating HCC cell migration may shed light to the development of novel targeted therapeutic intervention.

Deleted in liver cancer 1 (DLC1), a tumor suppressor gene, was first identified in primary HCC as a rat p122RhoGAP homolog [3]. In HCC, DLC1 has been found to possess tumor suppressive abilities [4]–[6] and is underexpressed mainly through gene deletion and DNA methylation [7]–[9]. Underexpression of DLC1 is also implicated in other cancers such as breast, lung, and prostate [9]–[14]. DLC1 was also shown to be downregulated in metastatic cells compared to non-metastatic cells in breast and HCC models [15], [16]. Ectopic expression of DLC1 was found to suppress cell migration and invasion in HCC, non-small cell lung cancer, breast cancer, lung cancer, ovarian cancer cell line models [4], [15], [17]–[21], and overexpression of DLC1 in metastatic breast cancer cell line could attenuate size and incidence of pulmonary metastases [15]. However, the molecular mechanisms underlying this suppression of cell movement and cancer metastasis remain unclear.

DLC1 is a member of the RhoGTPase activating protein (RhoGAP) family and possesses RhoGAP activity specific for RhoA [8]. DLC1 negatively regulates the activity of RhoA by enhancing intrinsic GTP hydrolytic activity of RhoA, thus catalyzing the conversion of RhoA from its GTP-bound active state to GDP-bound inactive state [22]. Rho-kinase (ROCK) is the best known downstream effector of RhoA [23], [24]. Binding of RhoA releases ROCK from its autoinhibitory structure and activates ROCK-mediated cellular events [23], [25]–[27]. ROCK is known to regulate cellular events related to cell motility. For example, ROCK was shown to control cell polarity by regulating PTEN/Akt signaling pathway in neutrophils [28] and control tail retraction in monocytes and prostate cancer cells [29]–[31]. ROCK also enhanced actomyosin contractility, a principal step of cell migration as described above [32]. Importantly, ROCK is a kinase that phosphorylates and activates many downstream substrates such as LIMK and myosin light chain 2 (MLC2). Phosphorylation of these substrates is important in regulating cytoskeletal reorganization and cell migration [33], [34]. Hyperactivation of the Rho/ROCK pathway is known to be associated with more aggressive tumor properties such as metastasis and invasion [35]–[41]. Deregulation of Rho/ROCK pathway may be consequentially related to its aberrant upstream regulatory pathway. We previously demonstrated that DLC1 is a RhoGAP protein and it is therefore logical to speculate that DLC1 suppresses cancer cell metastasis through negatively regulating ROCK-mediated cytoskeletal rearrangement. However, this hypothesis has never been tested experimentally and the mechanistic basis of how DLC1 suppresses cancer cell metastasis has not been delineated. Hence, it is strategic to examine the possible functional links between DLC1 and ROCK pathway and their implications in HCC.

In this study, we demonstrated that DLC1 inhibited ROCK-mediated cytoskeletal events including formation of stress fiber and focal contact network and phosphorylation of MLC2 at cell cortex. Significantly, these inhibitory effects of DLC1 depended on its RhoGAP activity. In addition, we have shown that DLC1 functioned as a negative regulator of ROCK in controlling cell morphology. Overall, we demonstrated that DLC1 negatively regulated ROCK in suppressing cell movement in HCC.

## Results

### DLC1 abolished formation of ROCK-mediated stress fiber and focal adhesion network

**Figure 1:**
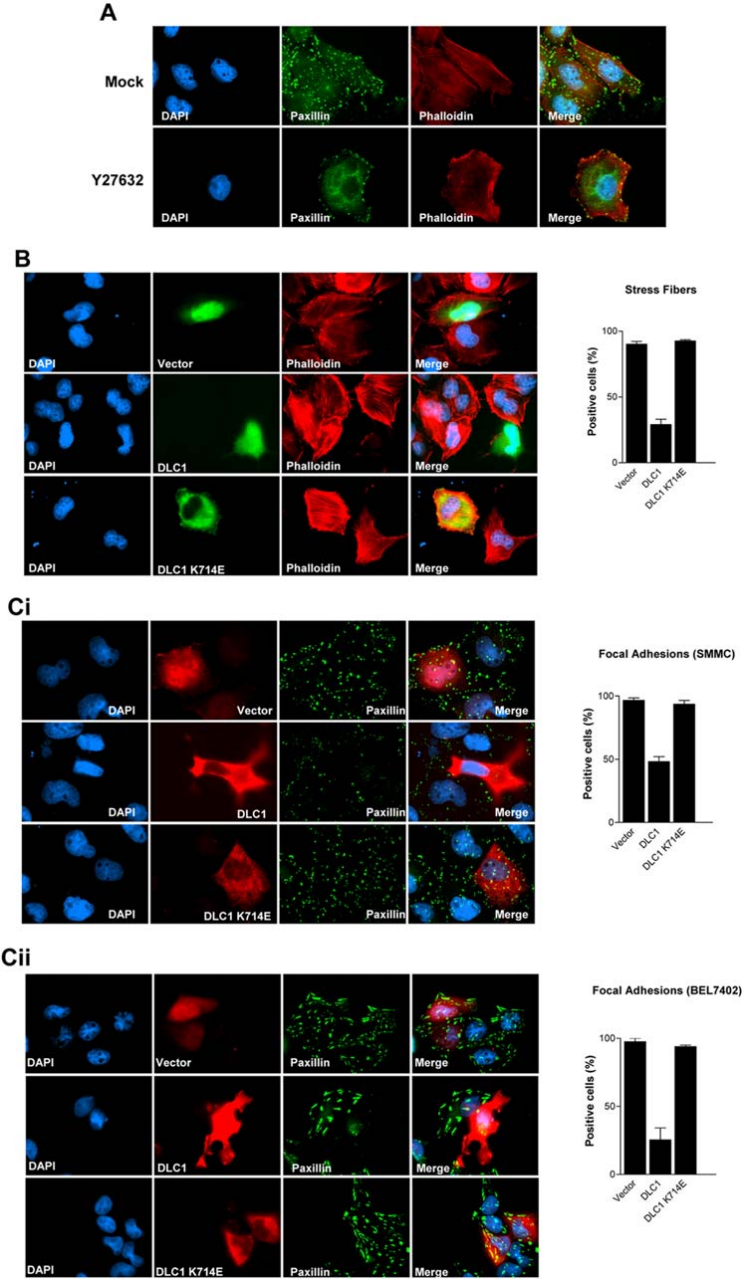
DLC1 abolished ROCK-mediated stress fiber and focal adhesion formation. (A) ROCK inhibitor suppressed stress fiber and focal adhesion formation in HCC cells. SMMC-7721 cells were seeded onto cover-slip one day before treatment. Stress fibers were stained with phalloidin stain (red) and focal adhesions with paxillin stain (green). Stress fibers could be clearly observed as bundles stretching across the cells and focal adhesions were attached to the stress fiber bundling arrays in SMMC-7721 mock treated control. Treatment with ROCK inhibitor Y27632 at 10 µM for 60 minutes suppressed formation of stress fiber and focal adhesion network in SMMC-7721. (B) and (C) DLC1 suppressed ROCK-mediated stress fiber and focal adhesion formation in HCC cells. SMMC-7721 cells (i) and BEL7402 (ii) were transfected with myc-tagged expression plasmids, pCS2+MT, pCS2+MT/DLC1, and pCS2+MT/DLC1 K714E, and recognized by anti-myc antibodies (9E10). Stress fibers and focal adhesions were stained with phalloidin-TRITC and anti-paxillin antibody, respectively. Wild-type DLC1, but not RhoGAP-deficient mutant (DLC1 K714E), suppressed stress fibers formation and reduced the number of stress fibers-linked focal adhesions formed in SMMC-7721 cells. Percentage of different DLC1 constructs transfected cells exhibiting stress fibers or focal adhesions were presented in a bar graph accordingly. For each construct, total 67–170 transfected cells were counted under microscope. Error bars represent standard deviation (SD) of data obtained from two independent experiments.

First, we queried if the formation of stress fibers and focal adhesions in HCC cells was ROCK dependent. We found that, the stress fiber bundling arrays (stained with phalloidin stain) and focal adhesions (paxillin), particularly those in the central region that were linked to stress fibers, were abolished upon ROCK inhibitor treatment (Figures 1A). This finding indicates that the formation of the stress fibers and focal adhesions in HCC cells is ROCK-dependent.

To investigate the regulatory function of DLC1 on ROCK-mediated stress fiber and focal adhesion formation in HCC, we transiently overexpressed DLC1 in DLC1-deficient SMMC-7721 and BEL7402 HCC cells and its effects on the network of stress fibers and focal adhesions were studied. Overexpression of wild-type DLC1 significantly suppressed formation of stress fibers and focal adhesions in these cells, as indicated by phalloidin (Figure 1B) and paxillin stains (Figure 1C-i and 1C-ii), respectively. Analogous to ROCK inhibition (Figure 1A), DLC1 abolished mainly the stress fiber-linked focal adhesions located in the central region of cells (Figure S1A). The loss of focal adhesions was further aggravated with ectopic expression of SAM domain-deleted mutant of DLC1 (ΔSAM) (Figure S1B), a DLC1 truncated construct that caused a more severe cell shrinkage and loss of stress fibers [4]. In contrast, a RhoGAP-deficient mutant (DLC1 K714E) was unable to inhibit both stress fiber and focal adhesion formation (Figures 1B and 1C). These findings demonstrated that DLC1 inhibited ROCK dependent stress fiber and focal adhesion formation via its RhoGAP activity.

### DLC1 abolished ROCK-mediated cortical myosin light chain 2 phosphorylation

**Figure 2:**
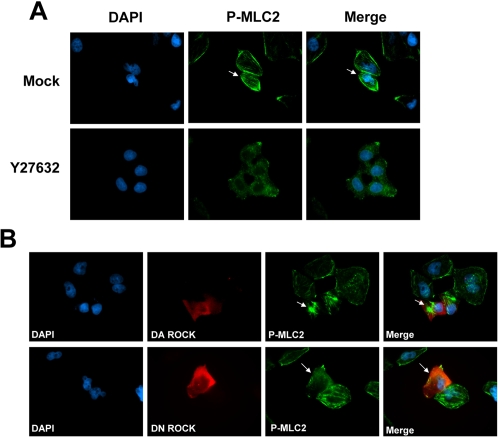
ROCK-mediated cortical phosphorylation of MLC2. (A) BEL4702 HCC cell line treated with mock control or ROCK inhibitor (Y27632) was probed with mouse monoclonal antibody against phospho-MLC2 (Ser 19). Phospho-MLC2 (green) was mainly detected at the cell cortex of BEL7402 cells (as indicated by the arrows) and this cortical staining pattern was abolished by treatment with ROCK inhibitor Y27632. (B) BEL7402 cells were transfected with myc-tagged dominant negative ROCK or dominant active ROCK constructs and detected with rabbit polyclonal antibody against myc-epitope (A14) (Red). Dominant active (DA) ROCK caused intense phosphorylation of MLC2 (Ser 19) at the actin bundles (Precentage of cells exhibiting this phenomenon: 94.0% of the DA ROCK-transfected cells compared to 0% of the vector transfected cells). Arrows point at the actin bundles of the dominant active ROCK transfected cell where phosphorylation of MLC2 (Ser 19) was enhanced. Dominant negative (DN) ROCK suppressed cortical phosphorylation of MLC2 (Ser 19) (Percentage of cells exhibiting this phenomenon: 87.5% of the DN ROCK-transfected cells compared to 7.3% of the vector transfected cells). Arrows point at the cell cortex of the dominant negative ROCK transfected cell where phosphorylation of MLC2 (Ser 19) was lost. As shown, a tight regulation and an optimal level of ROCK activity were required to maintain cortical phosphorylation of MLC2 (Ser 19) in BEL7402 cells.

Active ROCK specifically phosphorylates myosin light chain 2 (MLC2) at Ser 19; therefore, this ROCK-specific phosphorylation has been widely used as a surrogate marker of ROCK activity [36], [42]–[45]. Phosphorylation of MLC2 at Ser19 is important for the activity of myosin which is responsible for actomyosin contractility and hence cell migration [46]. In this study, we observed that phospho-MLC2 staining of BEL7402 HCC cells was especially prominent at the cell cortex. However, when ROCK activity was blocked either by Y27632 (Figure 2A) or ectopic expression of a dominant-negative ROCK mutant (Figure 2B), this peripheral phosphorylation of MLC2 was completely abolished and the phosphorylation of MLC2 became a predominantly cytoplasmic pattern. In contrast, ectopic expression of dominant-active ROCK culminated in a substantial increase of MLC2 phosphorylation at actin bundles (Figure 2B). These findings indicate that this distinctive phosphorylation pattern of MLC2 at cell cortex is tightly and positively regulated by ROCK activity.

**Figure 3:**
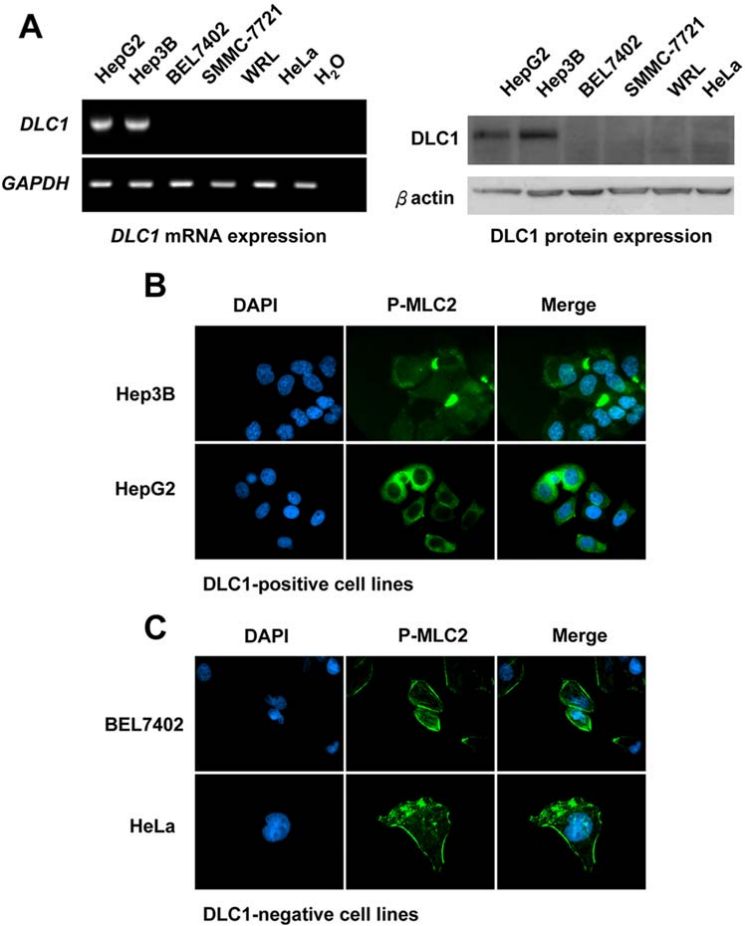
DLC1 expression was associated with cortical phosphorylation of MLC2. (A) DLC1 mRNA and protein expression in HCC cell lines. (B) and (C) DLC1 expression was associated with phospho-MLC2 staining pattern. Representative pictures from DLC1-positive and DLC1-negative cells, respectively. DLC1-positive Hep3B and HepG2, displayed diffuse phosphorylation of MLC2, whereas DLC1 non-expressing BEL7402 and HeLa, displayed pronounced cortical phosphorylation of MLC2 at cell cortex.

We next investigated whether DLC1 could abolish this ROCK-specific MLC2 phosphorylation pattern in HCC cells. We first evaluated whether the cortical phospho-MLC2 staining pattern was related to the endogenous expression levels of DLC1 in different cell lines. We observed conspicuous phospho-MLC2 staining at the cell cortex in SMMC-7721, BEL7402, and WRL HCC cell lines (Figure 3C), and HeLa cervical cancer cell line (Figure 3C), which had no expression of DLC1 (Figure 3A). On the other hand, HepG2 and Hep3B, the two HCC cell lines with DLC1 expression (Figure 3A), displayed diffuse cytoplasmic phospho-MLC2 staining without any distinct cortical phosphorylation of MLC2 at cell periphery (Figure 3B).

**Figure 4:**
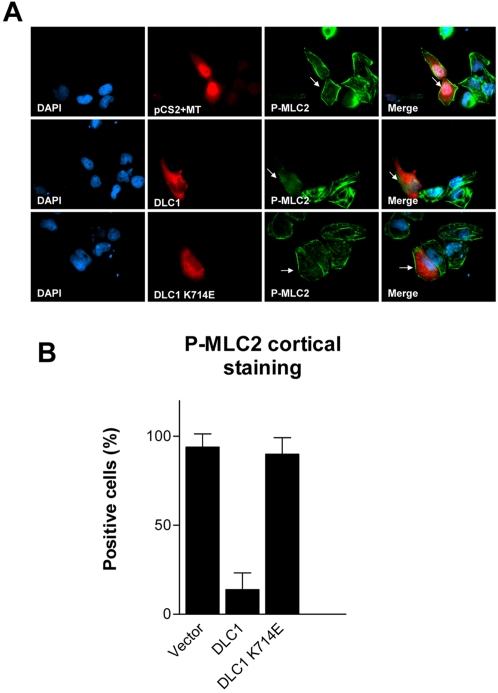
DLC1 RhoGAP was responsible for regulating cortical phosphorylation of MLC2. (A) pCS2+MT vector alone, pCS2+MT DLC1, and pCS2+MT DLC1 K714E, respectively, were transfected into BEL7402 cells. Recognition of transfected cells was done by probing cells with c-myc (A14) rabbit antibody followed by staining with anti-rabbit antibody conjugated with Texas Red. Cells transfected with pCS2+MT vector alone displayed pronounced cortical phosphorylation of MLC2 as indicated by the arrows. Cells transfected with DLC1 displayed loss of cortical phosphorylation of MLC2. Cells transfected with DLC1 K714E, the RhoGAP-deficient mutant, still displayed pronounced cortical phosphorylation of MLC2 as indicated by the arrows. (B) Percentage of different DLC1 constructs exhibiting cortical phosphorylation of MLC2 at Ser 19 was calculated and presented in a bar graph. For each construct, 80–100 transfected cells were counted and cortical MLC2 phosphorylation was recorded. Error bars represent standard deviation (SD) of data obtained from three independent experiments.

To verify that inhibition of cortical phospho-MLC2 was directly related to DLC1, we then transiently expressed wild-type DLC1 and DLC1 RhoGAP-deficient mutant (K714E), respectively, in DLC1-null BEL7402 cells. Wildtype DLC1 substantially reduced the number of cells having phospho-MLC2 cortical staining (Figures 4A & 4B), signifying the suppressive role of DLC1 on ROCK activity. On the other hand, DLC1 RhoGAP-deficient mutant (K714E) was unable to abolish phospho-MLC2 cortical staining (Figures 4A & 4B). Our observation therefore suggested DLC1 inhibited ROCK-specific MLC-2 phosphorylation in HCC cells, via its RhoGAP activity.

### DLC1 attenuated myosin phosphatase activity by reducing phosphorylation of myosin phosphatase target subunit 1 (MYPT1) at Thr 853

**Figure 5:**
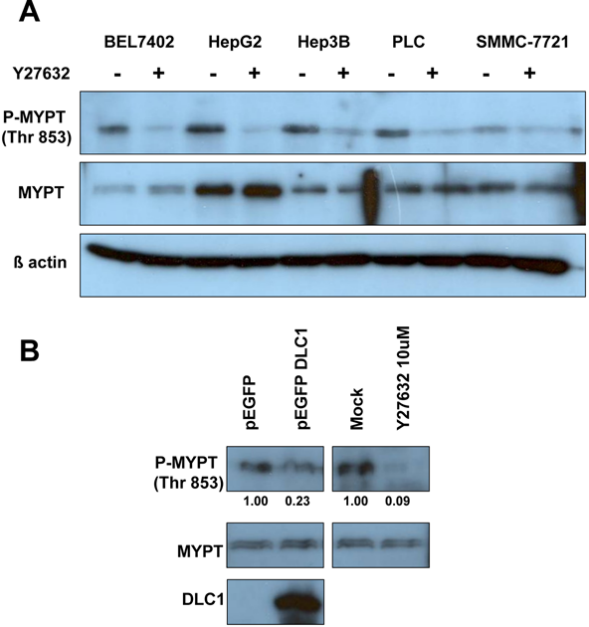
DLC1 and ROCK regulated MYPT phosphorylation. (A) ROCK inhibitor Y27632 suppressed MYPT phosphorylation at Thr 853 in all HCC cell lines. (B) DLC1 suppressed MYPT phosphorylation at Thr 853 in 293 T cells. Band intensity was analyzed by AlphaEaseFC™ and percentage was calculated from comparison with its according control.

Apart from phosphorylation of myosin, ROCK also increases myosin phosphorylation by phosphorylating myosin phosphatase target subunit 1 (MYPT1) at Thr 696 and Thr 853 and thereby inactivating it. Although Thr 696 of MYPT1 can be phosphorylated by other protein kinases, Thr 853 phosphorylation, on the other hand, is selectively regulated by ROCK [32]. Moreover, MYPT1 Thr 853 phosphorylation has been shown to be a promising surrogate marker that reversely correlates with myosin phosphatase activity [36], [42]–[45]. In this study, we found that, upon Y27632 treatment, the phosphorylation levels of MYPT1 (Thr 853) were reduced in all HCC cell lines tested (Figure 5A), while the total MYPT1 levels remained unchanged. Similarly, overexpression of DLC1 attenuated phosphorylation of MYPT1 at Thr853, indicating the loss of ROCK activity (Figure 5B). Inactivation of myosin phosphatase (as reflected by increased MYPT1 phosphorylation) and activation of MLC2 are combined effects of ROCK, and these effects could be suppressed by DLC1, leading to a total decrease of myosin phosphorylation and a reduction of cell contractility.

### ROCK inhibitor suppressed HCC cell motility

**Figure 6:**
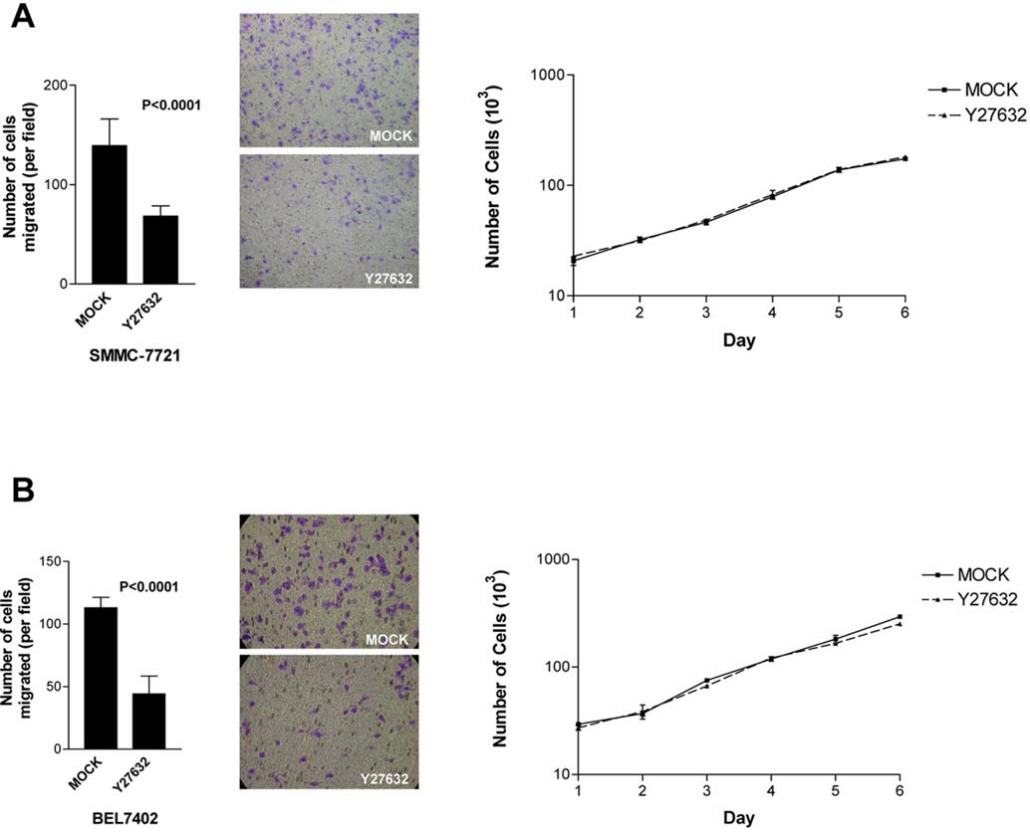
ROCK inhibitor suppressed HCC cell migration. ROCK inhibitor Y27632 suppressed HCC cell migration. Y27632 decreased number of migrated (A) SMMC-7721 cells (*P*<0.0001, *t*-test) and (B) BEL7402 cells (P<0.0001, *t*-test). Error bars represent standard deviation (SD) of data obtained from three microscopic fields. Experiments have been repeated three times. Cell proliferation assay was shown on the right to compare growth rate of mock control cells and Y27632 treated cells.

By ectopic expression of DLC1 in HCC cell line, we previously showed that DLC1 significantly repressed HCC cell migration and invasiveness [4]. To substantiate our hypothesis that DLC1 suppressed cell migration via negatively regulating ROCK activity, we examined the effect of ROCK inhibitor on HCC cell migration with transwell assay. We observed that the ROCK inhibitor Y27632 suppressed migration of HCC cells, SMMC-7721 (Figure 6A left panel) and BEL7402 (Figure 6B left panel), as demonstrated by the decreased number of migrated cells in transwell assay. This finding indicates that ROCK activity is crucial for HCC cell migration. To eliminate any misinterpretation due to cell death caused by toxicity of the drug, we performed cell proliferation assay on the HCC cells. Cell proliferation assay demonstrated that Y27632 did not affect cell growth which confirmed that inhibition of ROCK only affected HCC cell migration (Figure 6A and 6B right panel).

### ROCK reversed the cell morphological alteration of DLC1

**Figure 7:**
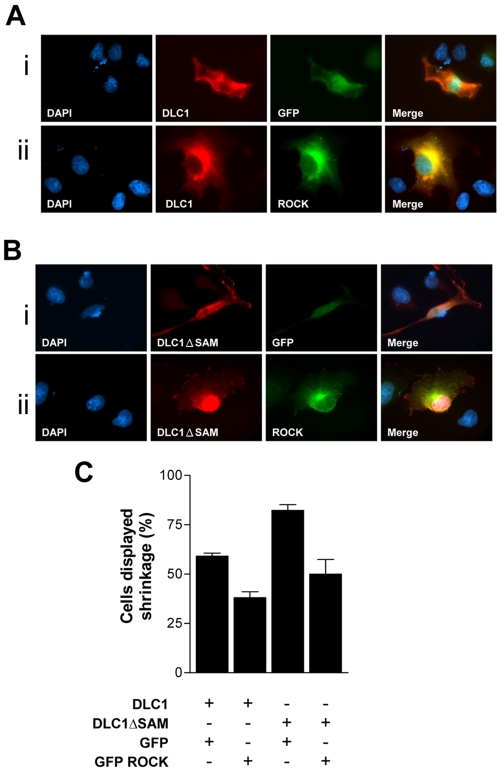
ROCK reversed the cell morphological alteration of DLC1. COS7 cells were co-transfected with (A) DLC1 and ROCK or (B) DLC1ΔSAM and ROCK, respectively. Cells transfected with pCS2MT DLC1 or pCS2MT DLC1ΔSAM appeared red, while cells transfected with pEGFP or pEGFP ROCK appeared green. DLC1-induced cell cytoskeletal collapse or cell shrinkage (A-i), which was further enhanced and clearly demonstrated by SAM-deleted construct, pCS2+MT DLC1ΔSAM (B-i). ROCK was able to restore cells from DLC1-induced cell shrinkage (A-ii) and even from DLC1ΔSAM-induced intense cell shrinkage (B-ii). (C) Co-transfected cells were counted and their morphologies recorded. The number of co-transfected cells displaying observable cell shrinkage (cell collapse) was divided by total number of co-transfected cells counted, to calculate the percentage of cells in displaying shrinkage as shown in the bar graph. For each column, 150–200 co-transfected cells were counted. Error bars represent standard deviation (SD) of data obtained from three independent experiments.

We previously demonstrated that DLC1 was able to induce cell morphological change with reduced stress fiber formation in HCC cells and fibroblasts [4]. Since ROCK is a chief modulator in cytoskeletal signaling and cytoskeletal organization is a key determinant of cell morphology, we speculated that the cell morphological changes induced by DLC1 were via ROCK. To this end, we observed the cell morphology of DLC1 and ROCK co-transfected cells by immunofluorescence staining. We also counted the number of cells that exhibited cell collapse or shrinkage caused by collapse of cytoskeletal network (Figure 7C), as described elsewhere [4], [47]–[49]. In this study, we observed that overexpression of DLC1 in COS7 cells induced cytoskeletal collapse or cell shrinkage (Figure 7A-i), similar to our previous finding [4]. Cell shrinkage was intensified by ectopic expression of SAM domain-deleted mutant of DLC1 (ΔSAM) (Figure 7B-i). Co-transfection of empty pEGFP vector could not rescue DLC1-induced cell morphological change, but cells with dominant active ROCK could overcome the inhibitory regulation from DLC1 (Figure 7A-ii) and even DLC1ΔSAM (Figure 7B-ii) and released cells from DLC1-induced cell shrinkage. This experiment showed that ROCK is one of the downstream effectors of DLC1 in coordinating the morphological changes in HCC cells.

## Discussion

One of the important roles of DLC1 is its ability in repressing cancer cell migration and invasion. In this study to elucidate the underlying mechanisms in the regulation of HCC cell migration and invasion by DLC1, we have shown that DLC1 suppressed HCC cell migration through regulating the cytoskeleton and actomyosin contraction. Also, we have shown that DLC1 functioned as a negative regulator of ROCK in controlling cell morphology.

### DLC1 negatively regulated ROCK mediated actomyosin contractility

The contractile motion of cancer cells is generated by consecutive contractions of actomyosin bundles composed of actin and myosin called actomyosin. These actomyosin bundles, visualized as stress fibers, stretch across the cell body and create a tension to generate cell contraction to drive cell movement by connecting with the focal adhesion molecules [50]. Based on the previous study on the inhibitory effect of DLC1 on stress fibers, herein we further found that the formation of stress fibers and focal adhesions network was dependent on DLC1 RhoGAP and ROCK activity in HCC cells. This finding further elucidates that stress fibers and focal adhesions work hand in hand as described by Hall A. [51], and their formation is interdependent and tightly regulated by RhoGAP of DLC1 and ROCK activity. In addition, DLC1 decreased ROCK-mediated phosphorylation of MYPT1 (Thr853) and phosphorylation of MLC2 (Ser19) and would consequently lead to a decrease of total myosin phosphorylation and cause a reduction of stress fiber contractility [46]. All these lines of evidence converged to explain that DLC1 controlled one of the pivotal migratory mechanisms, actomyosin contraction, similar to another member of the RhoGAP family, p190-RhoGAP [52].

### DLC1 abolished focal adhesion formation and also localized to focal adhesion complex

An intriguing issue of this finding about the inhibitory effect of RhoGAP on focal adhesion molecules has arisen. DLC1 and several members of the RhoGAP proteins including p122-RhoGAP and RC-GAP72 were found to be localized at the focal adhesions [47], [49]. In fact, others and we have previously reported that DLC1 also localized to focal adhesions and interacted with members of the tensin family [18], [53], [54]. In this study, we found that DLC1 suppressed stress fiber-linked focal adhesion formation by its RhoGAP activity. From immunofluorescence study, we observed that, in a portion of DLC1 transfected cells, DLC1 exhibited a focal adhesion localization pattern (Figure S1A); whereas in some other DLC1 transfected cells, especially those displaying extensive cell shrinkage, an intensive loss of stress fiber-linked focal adhesions was observed (Fig. 1C). DLC1 localization to focal adhesions and its inhibitory effect on focal adhesions are not mutually exclusive. We speculate that dosage effect might be one of the factors involved. When a cell was transfected with a high dose of DLC1, DLC1 would extinguish all the stress fibers and the stress fiber-linked focal adhesions and result in extensive cell collapse (Fig. 1C). On the other hand, when a cell was transfected with a lower dose of DLC1, DLC1 would extinguish most of the stress fibers and stress fiber-linked focal adhesions but spare some focal adhesions not linked to stress fibers and result in a milder cell collapse (Figure S1A). Another speculation was that similar to RC-GAP72 [47], DLC1 might localize to focal adhesion to disintegrate the focal adhesion complexes.

### Cytoskeletal collapse induced cell shrinkage and the impact of DLC1 on cytoskeletal collapse

**Figure 8:**
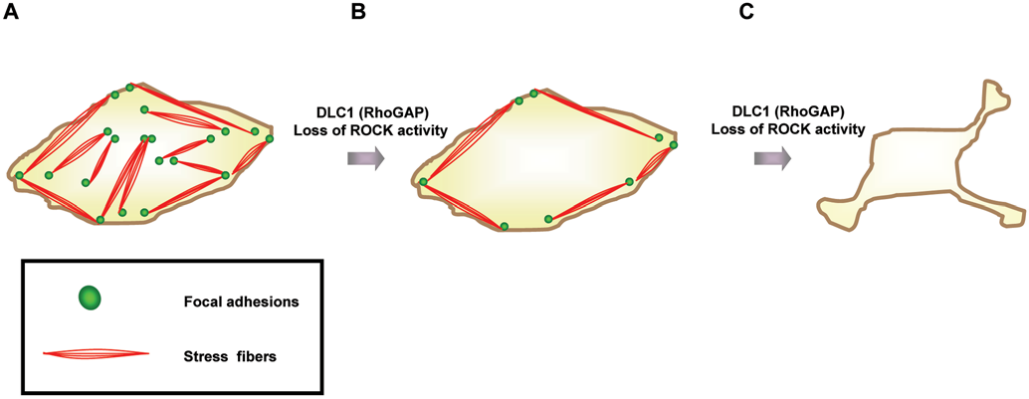
Model of DLC1 induced cytoskeletal collapse. (A) Stress fibers were connected to focal adhesions forming a network. Stress fibers stretched across the cell to provide the cell an intact morphology. (B) DLC1 RhoGAP or loss of ROCK activity induced the loss of stress fibers and focal adhesions. A few focal adhesions and bundles of stress fibers remained. (C) A prolonged or severe loss of stress fiber and focal adhesion network caused by DLC1 RhoGAP or suppression of ROCK activity would result in intensive cytoskeletal collapse. All the stress fibers and focal adhesions were abolished.

Focal adhesions and actomyosin stress fibers always work in inter-dependent manner and they together construct a scaffold to support an intact morphology of the cell [47]. The disruptions of the abovementioned network caused by DLC1 led to an intensive cell cytoskeletal collapse resulting in cell shrinkage. This finding was similar to Barberis D *et al.*'s study on p190RhoGAP that loss of focal adhesions induced by p190RhoGAP preceded cell shrinkage [48]. We observed that loss of focal adhesions and stress fibers took place an hour after ROCK inhibition by Y27632, but cells did not collapse immediately (Figure 1A) until prolonged treatment of Y27632 up to 24 hours (Figure S2). From this observation, we conjecture that cell collapse caused by Y27632 treatment was ensued from loss of focal adhesion and stress fiber network. Upon inhibition of ROCK by Y27632, the cytoskeletal networks could not be formed and the cells could no longer endure the loss of cytoskeletal network and eventually undergo cell collapse, which we observed as cell shrinkage (Figure 8). The present study showed that the cytoskeletal collapse induced by DLC1, a RhoGAP which its downregulation is associated with HCC progression, could be partially reversed by active ROCK, further implying that DLC1 negatively regulated ROCK in controlling actomyosin contractility, cell morphology, and cell migration sequentially. Our previous study reported that DLC1 not only suppressed cell migration but also cell invasion which involved extracellular matrix barrier. Sahai E *et al.* reported that cancer cell lines might possess different modes of cell motility and the rounded morphology cancer cell lines were more sensitive to ROCK inhibitor in 3-dimensional matrix [55]. However, whether this model is applicable to HCC cells and whether other extracellular proteolytic pathways coorperate with the DLC1/Rho/ROCK/MLC pathway are still unknown and awaited to be addressed.

Our study demonstrated that ROCK inhibitor, Y27632, did not affect HCC cell proliferation at an efficient dosage that suppressed HCC cell migration. ROCK inhibitors have been widely used in animal models, such as rat and mice models, without causing major toxicity to the animals at efficient dosages [35], [56]. Takamura et al. demonstrated that Y27632 could inhibit intrahepatic metastasis of HCC [35] and recently, another ROCK inhibitor, fasudil, has been successfully applied in clinical trial for patients with cardiovascular diseases [57]. These studies demonstrate that the toxicity of ROCK inhibitors to cells is limited [35]. Accumulating knowledge indicates that ROCK is playing an important role in cancer metastasis, and the present study has enriched the knowledge on the ROCK signaling pathway in HCC. ROCK inhibitors such as Y27632 might be useful to the chemotherapeutic intervention in suppressing intrahepatic metastasis, a major cause of mortality in HCC patients, without causing much adverse effects to the patients. Further characterization of the efficacies of ROCK inhibitors and anti-ROCK therapies in the treatment of HCC is still awaited.

## Materials and Methods

### Cell lines and plasmids

SMMC-7721 and BEL7402 were gifts from Shanghai Institute of Cell Biology, Chinese Academy of Sciences; 293T, COS7, HepG2 and Hep3B were obtained from American Type Culture Collection (Manassas, VA). 293T, BEL7402, SMMC-7721, and COS7 were cultured in Dulbecco's Modified Eagle Medium (DMEM) high glucose supplemented with 10% fetal bovine serum; Hep3B and HepG2 were cultured in minimum essential medium (MEM) supplemented with 10% fetal bovine serum. Myc-tagged expression constructs (pCAG) carrying human wild-type ROCK, dominant active ROCK (1–725 a.a.), and dominant negative ROCK mutant (K105A, I1009A) were kindly provided by S. Narumiya (Faculty of Medicine, Kyoto University) [27]. The human ROCK kinase domain (ROCK 76–338 a.a.) was PCR amplified and cloned into pEGFPC3 vector using KpnI and XhoI digestion sites and was used as dominant active form of ROCK. Wild-type DLC1 and RhoGAP-deficient mutants were cloned as described previously [4].

### Drug treatment and transfection

ROCK-specific inhibitor Y27632 was obtained from Calbiochem (Darmstadt, Germany). For treatment, cells were added Y27632 at 10 µM and incubated for 1 hour at 37°C. For transfection, 1×10^5^ cells were seeded on coverslips in 35-mm plates one day before transfection. Indicated plasmids were transfected into cells with FuGene 6 reagent (Roche, Basel, Switzerland) according to manufacturer's instructions.

### Immunofluorescence microscopy

Mouse monoclonal antibody against phospho-myosin light chain 2 (Ser 19) was purchased from Cell Signaling Technology (Danvers, MA). Mouse monoclonal antibody against paxillin was obtained from Upstate (Lake Placid, NY). Mouse monoclonal (9E10) and rabbit polyclonal (A14) antibodies against c-myc were obtained from Santa Cruz Biotechnology (Santa Cruz, CA). Texas Red dye-conjugated AffiniPure goat anti-mouse IgG and fluorescein (FITC)-conjugated AffiniPure goat anti-rabbit IgG were purchased from Jackson Immuno Research Laboratories (West Grove, PA). For immunofluorescence staining, cells were fixed in paraformaldehyde, permeabilized with TritonX, blocked with bovine serum albumin and then incubated with indicated primary and secondary antibodies. Focal adhesions were probed with anti-paxillin antibody and stress fibers were stained with phalloidin TRITC (Sigma Aldrich, St. Louis, MO). Nuclear counterstaining was done with 4′-6-Diamidino-2-phenylindole (DAPI) (Calbiochem, San Diego, CA) and coverslips were mounted in Vectashield antifade mountant (Vector Laboratories, Burlingame, CA). Cells were counted under Leica Q550CW fluorescence microscope (×1,000 magnification) and images were captured with a charge-coupled device camera connected to the microscope.

### Cell migration assay

Transwell assay was performed with Transwell Boyden chamber of polycarbonate membranes with pore size of 8.0 µm (Corning Inc., NY). 5×10^4^ cells resuspended in serum free culture medium were added to the upper chamber whilst culture medium with 10% FBS was placed in the lower chamber as chemoattractant. Cells were incubated in CO_2_ incubator at 37°C. Cells having migrated through the pores to the lower surface of the membrane were fixed with methanol and stained with crystal violet. Photographs of 3 different fields of the stained cells were captured and cells were counted. Experiment was repeated independently three times.

### Cell proliferation assay

2×10^4^ cells were seeded in 12 well plates. Cells were counted everyday with COULTER COUNTER® Cell and Particle Counter (Beckman Coulter) in triplicates and a 6-day growth curve was plotted. Cells were replenished with fresh medium (mock or Y27632) every other day.

### mRNA extraction, protein extraction and western blots

Total RNA was extracted by TRIZOL reagent, cDNA was synthesized, and the expression of DLC1 mRNA and GAPDH was detected by RT-PCR with specific primers as described previously [8]. For Western blot, cells were lysed with SDS buffer or NP40 in NET buffer. Proteins were resolved by SDS-PAGE and blotted onto a nitrocellulose membrane. The membrane was probed using anti-DLC1 antibody (BD Biosciences Pharmingen), anti-MYPT antibody or anti-phospho-MYPT (Thr853) antibody (Upstate), followed by incubation with anti-mouse IgG or anti-rabbit IgG (GE Healthcare, Buckinghamshire, UK). Protein expression was detected with the ECL™ detection system (GE Healthcare) according to the manufacturer's protocol. Images were scanned and intensity of bands was quantified with AlphaEaseFC™ software.

## Supporting Information

Figure S1 Effect of DLC1 on focal adhesions.
(0.28 MB PDF)

Figure S2 Prolonged ROCK inhibitor treatment induced HCC cell collapse.
(0.29 MB PDF)
