# Supplementary material for: Deleted in Liver Cancer 1 (DLC1) Negatively Regulates Rho/ROCK/MLC Pathway in Hepatocellular Carcinoma
Source: PLoS One. 2008 Jul 23;3(7):e2779. doi: 10.1371/journal.pone.0002779 (PMC2464714; doi:10.1371/journal.pone.0002779)
Supplement: Figure S1 — Effect of DLC1 on focal adhesions. (0.28 MB PDF) [file pone.0002779.s001.pdf]

Supplementary Figure 1A (S1A)

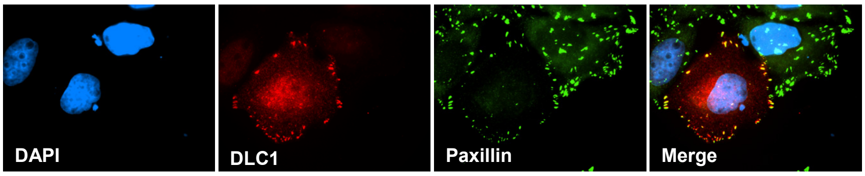

A portion of the cells transfected with DLC1 displayed a loss of stress fibers-linked focal adhesions; meanwhile, DLC1 colocalized to the remaining adhesions. Unlike many of the cells transfected with DLC1 that displayed a total loss of focal adhesions, cells displaying this phenomenon appeared with a less severe cell shrinkage.

Supplementary Figure 1B (S1B)

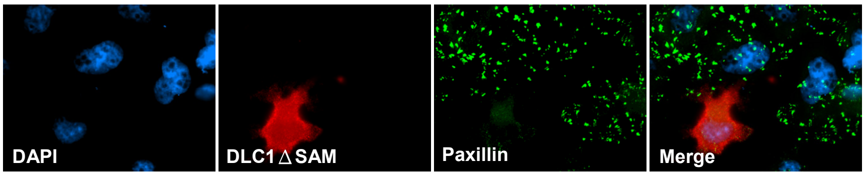

Cells transfected with DLC1 △SAM displayed an intensive cell shrinkage and a loss of focal adhesions. Loss of focal adhesions is believed to be associated to cell shrinkage because cells with more severe cell shrinkage displayed more severe loss of focal adhesions.
