# Supplementary material for: Deleted in Liver Cancer 1 (DLC1) Negatively Regulates Rho/ROCK/MLC Pathway in Hepatocellular Carcinoma
Source: PLoS One. 2008 Jul 23;3(7):e2779. doi: 10.1371/journal.pone.0002779 (PMC2464714; doi:10.1371/journal.pone.0002779)
Supplement: Figure S2 — Prolonged ROCK inhibitor treatment induced HCC cell collapse. (0.29 MB PDF) [file pone.0002779.s002.pdf]

Supplementary Figure 2 (S2)

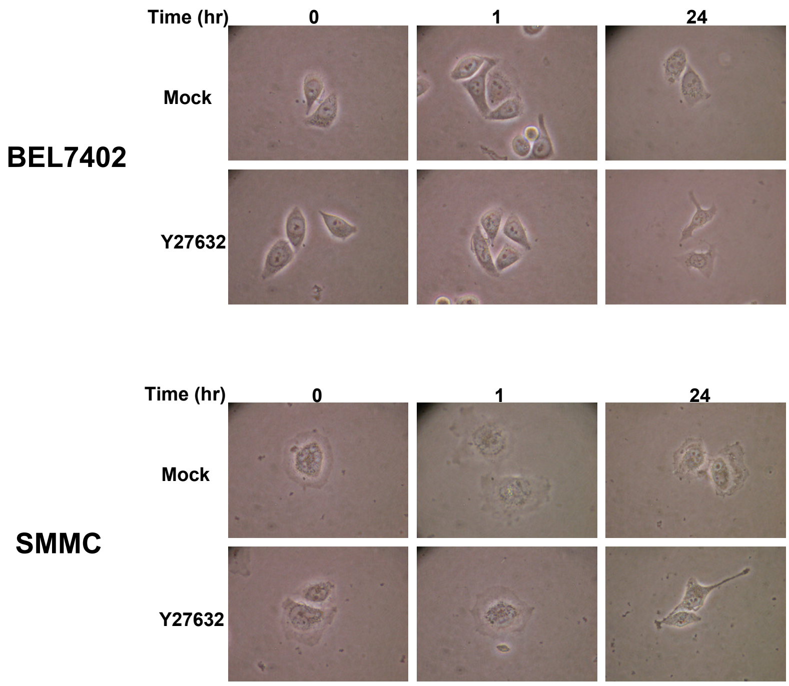

Prolonged ROCK inhibitor treatment induced HCC cell collapse. HCC cells, BEL7402 and SMMC, were treated with ROCK inhibitor, Y27632, as the indicated period of time. Short term inhibition of ROCK (1 hour) did not cause cell collapse in both HCC cell lines. Long term inhibition of ROCK (24 hours) by Y27632 caused cell collapse as shown in the figure.
